# New Frontiers in Functional and Molecular Imaging of the Acutely Injured Lung: Pathophysiological Insights and Research Applications

**DOI:** 10.3389/fphys.2021.762688

**Published:** 2021-12-09

**Authors:** Guido Musch

**Affiliations:** Department of Anesthesiology and Perioperative Medicine, University of Massachusetts Medical School, Worcester, MA, United States

**Keywords:** positron-emission tomography, tomography X-ray computed, magnetic resoance imaging, ventilator-induced lung injury, acute lung injury, respiratory distress syndrome, respiratory physiological phenomena, isotopes

## Abstract

This review focuses on the advances in the understanding of the pathophysiology of ventilator-induced and acute lung injury that have been afforded by technological development of imaging methods over the last decades. Examples of such advances include the establishment of regional lung mechanical strain as a determinant of ventilator-induced lung injury, the relationship between alveolar recruitment and overdistension, the regional vs. diffuse nature of pulmonary involvement in acute respiratory distress syndrome (ARDS), the identification of the physiological determinants of the response to recruitment interventions, and the pathophysiological significance of metabolic alterations in the acutely injured lung. Taken together, these advances portray multimodality imaging as the next frontier to both advance knowledge of the pathophysiology of these conditions and to tailor treatment to the individual patient’s condition.

## Introduction

Most reviews of imaging for acute lung injury start from the specific method and then derive, based on its technical and physical properties, the corresponding application. The approach of this review will be different. It will start from fundamental pathophysiologic and metabolic hallmarks of ventilator-induced and acute lung injury to derive the corresponding phenotypic trait that can be leveraged as an imaging target. Not aiming to be an exhaustive review of this topic, for which there are recent comprehensive papers (Cereda et al., 2019), the emphasis herein will be on phenotype-based imaging approaches that hold promise of significant new developments and applications as well as on the insights that such approaches have already yielded.

## Heterogeneous Loss of Aeration and Concentrated Lung Strain

Since the late 1980s, it became clear that what had been previously considered a homogeneous loss of aeration throughout the lung of patients with acute respiratory distress syndrome (ARDS) was instead heterogeneously distributed. Such loss involved only certain portions of the lung, most frequently the dependent, dorsal, and caudal regions, while often sparing the more anterior nondependent ones (Puybasset et al., 1998). A functional consequence of this heterogeneous loss of aeration is that the amount of lung available to accommodate tidal volume is reduced and hence potentially exposed to greater mechanical strain during ventilation (Gattinoni and Pesenti, 2005). Because the lung, in contrast to solid organs like the liver or the brain, contains gas, mechanical strain is accompanied by a change in gas volume and thus lung density and electrical impedance. This property has been leveraged by, respectively, computed and electrical impedance tomography to measure the regional distribution of tidal volumetric strain in mechanically ventilated patients with, and animal models of, ARDS. Computed tomography (CT) measures the *x*-ray absorbance of each “piece” (voxel) of lung, which is inversely proportional to its gas content. Multi-detector CT scanners allow coverage of the entire lung fields at very high spatial resolutions of 1–2 mm. This enables reconstruction of a detailed three-dimensional map of the distribution of lung gas volume vs. tissue volume. By taking CT scans at different phases of the respiratory cycle, such as end expiration and end inspiration, or at different end-expiratory pressures, one can derive information on the distribution of both static and dynamic lung strain. While CT has the definite advantage of a very high spatial resolution, its main limitations are: cost, radiation exposure, and extreme difficulty, if not inability, to use it as a bedside tool to guide ventilator strategy. Electrical Impedance Tomography (EIT) partially obviates these limitations, albeit at the expense of a much lower spatial resolution. In EIT, an electrode belt is placed around the chest and used to record cross-sectional voltages after stimulation with low-amperage alternating current. Changes in thoracic electrical impedance are related to changes in the amount of gas relative to lung tissue, edema, or blood volume, and hence have been used mainly to assess the regional distribution of ventilation and end-expiratory lung gas volume. A distillate of the findings of literally hundreds of studies performed with CT and EIT is:

- Loss of aeration is predominant in dependent dorsal and caudal regions (Puybasset et al., 1998).
- As a result, tidal volume distributes preferentially to the non-dependent ventral regions (Gattinoni et al., 1995; Spinelli et al., 2021), exposing them to increased and potentially injurious strain, which compounds the inflammatory process of ARDS and leads to ventilator-induced lung injury (VILI).
- The increased vertical pleural pressure gradient of the edematous lung defines a transition zone of poorly aerated lung tissue that undergoes the greatest cyclical changes in lung density during the respiratory cycle. This zone is thought to act as a focus of propagation of subsequent injury (Cereda et al., 2017; Xin et al., 2018)
- Recent evidence indeed suggests that lung regions that exhibit the greatest cyclical change in density with tidal volume at the start of a period of mechanical ventilation eventually become “injured” as defined by a stable increase of their density above −300 Hounsfield Units (Cereda et al., 2017);
- All of the above phenomena can be partially reversed with positive end-expiratory pressure (PEEP) or prone positioning (Gattinoni et al., 1995; Cornejo et al., 2013; Xin et al., 2018).

## Tomographic Imaging of Inhaled Gases To Derive Measures of Regional Ventilation and Alveolar Geometry

The above measurements of lung gas content with an external source be it of radiation or electricity, cannot directly trace the transport and distribution of inhaled gases to the alveolar airspace where gas exchange occurs. Several techniques were developed to overcome this limitation by administering inhaled gases that would yield a signal to be imaged. Herein, we will review three such techniques that hinge on three different imaging modalities, and the insights they provided.

Xenon is a radiodense gas and can thus be used as an inhaled contrast agent to study regional gas transport, ventilation, and lung strain with CT. Using this technique, Herrmann et al. (2021) recently demonstrated that multifrequency oscillatory ventilation, a new approach to oscillatory ventilation that uses more than one frequency in the ventilatory waveform, achieved fast gas transport rates, similar to conventional mechanical ventilation, but with much lower delivered volumes, comparable to high frequency oscillatory ventilation. This mode should thus afford the lower lung stretch of high frequency oscillation, while compensating the drawback of less efficient gas transport than conventional mechanical ventilation.

Another approach is to administer an inhaled radioactive gas, either a single photon, like krypton-81, or positron, like nitrogen-13 (^13^N_2_), emitter. The washin, equilibration, and washout kinetics can then be imaged by single photon emission computed tomography (SPECT) or positron emission tomography (PET), respectively. Using inhaled ^13^N_2_, Wellman et al. (2014) showed that high PEEP decreases tidal strain in middle and dependent lung regions of mechanically ventilated sheep exposed to intravenous lipopolysaccharide, an experimental model for sepsis. Inhaled gas methods have also allowed validation of strain measurements derived from lung density changes against other measurements of regional mechanics such as specific ventilation (Wellman et al., 2010) and parenchymal marker displacement (Fuld et al., 2008).

**Figure 1:**
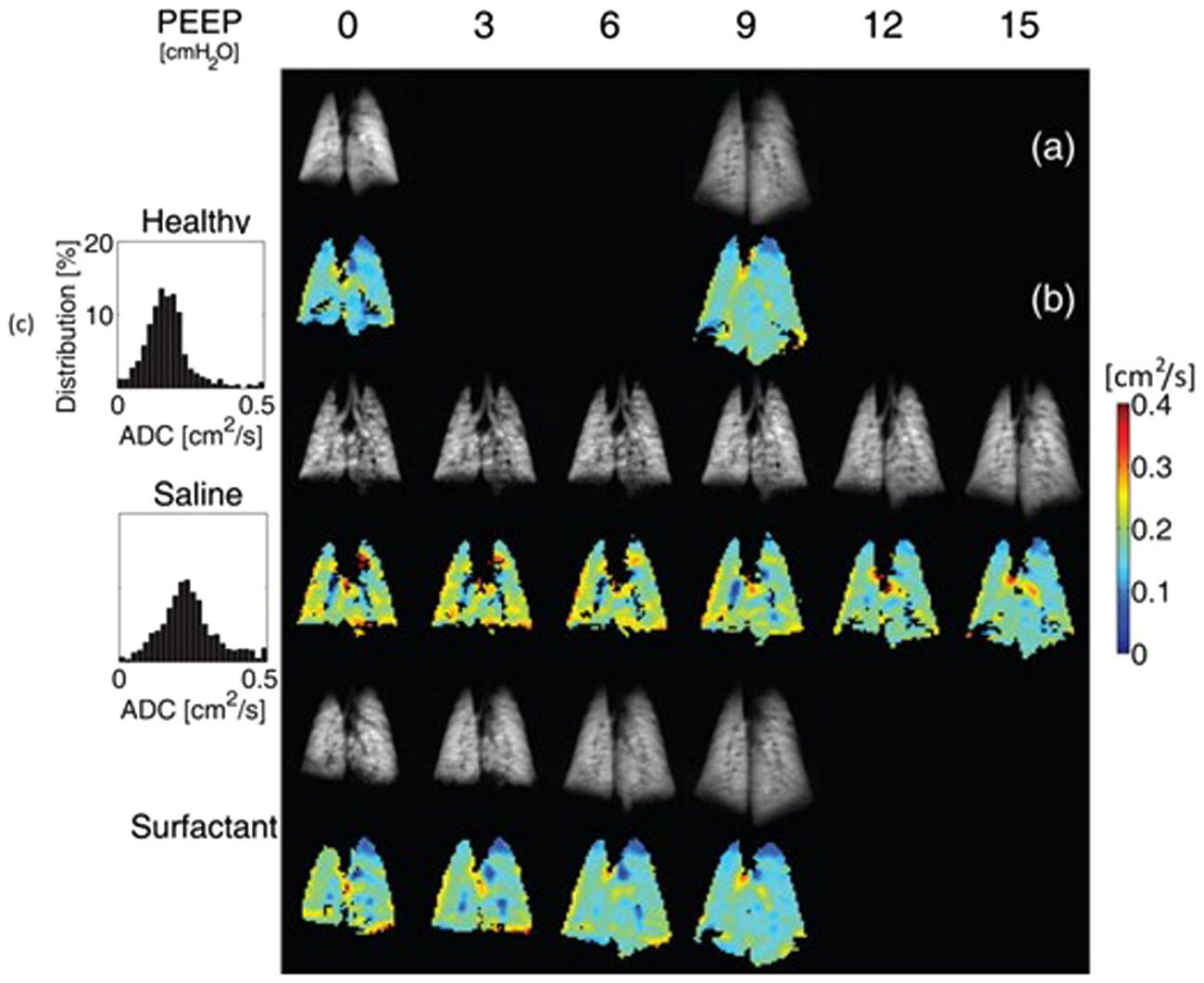
Effect of positive end-expiratory pressure (PEEP), surfactant depletion by saline lavage, and exogenous surfactant administration on helium-3 (^3^He) spin density **(A)** and alveolar size as inferred by apparent diffusion coefficient (ADC) for ^3^He **(B)** in a coronal slice of rat lung. At PEEP = 0 cmH_2_O, saline lavage shifted the ADC distribution toward higher values and increased its variance **(C)**, consistent with greater heterogeneity of aerated acini’s size and alveolar overdistension, as it can be appreciated by yellow and red speckling on the lung ADC map. PEEP and surfactant reduced this speckling, both individually and through a combined effect that resulted in restoration of an ADC map similar to the healthy state (Modified from Cereda et al., 2013a).

A third approach is based on inhalation of hyperpolarized gases, in particular helium-3 (^3^He) and xenon-129 (^129^Xe), the distribution of which can be imaged with magnetic resoance imaging (MRI). ^3^He has been used to measure the apparent diffusion coefficient (ADC) in models of ARDS and atelectasis during mechanical ventilation (Cereda et al., 2011, 2013a,b, 2016). Helium’s small nucleus enables rapid diffusivity, which is limited only by the restriction imposed by the alveolar wall. The ADC is a measure of how freely and far a nucleus can diffuse within a specific medium. This diffusivity is decreased by barriers that do not allow free passage of the nucleus, as are the alveolar walls in the lung. Consequently, the smaller is the size of the acinar space, the more restricted is the movement of ^3^He and the lower is the ADC. This technique has thus been particularly valuable to yield insights on airspace size changes with atelectasis and PEEP. In particular, this technique has led to an appreciation of the importance of alveolar interdependence when considering the effects of alveolar overdistension and derecruitment (Cereda et al., 2011). Whereas classical thinking had these two phenomena occurring at high and low extremes of airway pressure, respectively, and in topographically distinct parts of the lung (non-dependent vs. dependent), ^3^He MRI studies revealed that ADC of ventilated airspaces increases during ventilation at low airway pressure that promotes atelectasis, that this increase is reversed by alveolar recruitment maneuvers (Cereda et al., 2011), and that the highest ADC increase tends to colocalize with the loss of aeration in dependent dorsal regions (Cereda et al., 2016). In a saline-lavage model of ARDS (Cereda et al., 2013a), surfactant depletion resulted in a wider distribution of ADC, shifted toward higher ADC values. Combined application of PEEP and exogenous surfactant restored ADC values and distribution similar to those before lavage (Figure 1). In another study, application of PEEP to initially healthy but derecruited lungs resulted in reversal of ADC-hysteresis, with smaller ADC values on the descending than on the ascending limb of the pressure-ADC curve as opposed to the whole lung pressure-volume curve (Cereda et al., 2013b). Taken together, these results have been interpreted to indicate that alveolar derecruitment leads to overstretching of airspaces that remain open, due to alveolar interdependence. Interalveolar traction forces between open and atelectatic airspaces in the same or contiguous regions are relieved by alveolar recruitment, which results in a greater number of open airspaces, with consequent reduction of the forces and smaller and more homogenous distribution of alveolar size.

## Tomographic Imaging of Shunt, Edema, and Vascular Permeability

A pathophysiological hallmark of ARDS is increased pulmonary vascular permeability, which leads to interstitial and intraalveolar edema. The functional consequence of edema is shunt, whereby gas exchange with pulmonary capillary blood is impeded. These physiological alterations, and their topographical distribution, can be measured with PET of several isotopes. The key concept here is that the delivery of the tracer by inhalation is not useful because, by definition, inhaled tracer cannot reach regions of shunt, which are not ventilated. Therefore, even techniques like ^129^Xe MRI, which can measure gas transport efficiency in healthy lungs from the loss of ^129^Xe signal to the blood phase as Xe is absorbed into the pulmonary circulation (Ruppert et al., 2019), do not yield a measurable signal in shunting regions.

One approach to circumvent this roadblock is to administer a gaseous tracer in saline solution intravenously, so that it can reach shunting regions, which are perfused. Historically, the first gas used for this purpose was sulfur hexafluoride, as part of the multiple inert gas elimination technique. A necessary prerequisite for measuring “true” shunt is that such gas must have very low solubility in blood so that it entirely diffuses into the gas phase of non-shunting units at first pass, and is retained only in shunting units. A gas that has similarly low solubility and also the advantage of a positron emitting isotope is nitrogen. The corresponding PET technique to measure regional pulmonary perfusion and shunt is based on the intravenous administration of [^13^N]nitrogen (^13^N_2_) in saline solution (Galletti and Venegas, 2002; Vidal Melo et al., 2003). A bolus of ^13^N_2_ gas dissolved in 20–30 ml of saline is infused intravenously at the beginning of a 30- to 60-s apnea while the pulmonary kinetics of ^13^N_2_ is measured with sequential PET frames. Because of the low solubility of nitrogen in blood and tissues (partition coefficient between water and air is 0.015 at 37°C); virtually all infused ^13^N_2_ diffuses into the airspace of aerated alveoli at first pass, where it accumulates in proportion to regional perfusion (Musch et al., 2002). However, if alveoli are perfused but not aerated, for example because they are atelectatic or flooded with edema, ^13^N_2_ kinetics shows an early peak of tracer activity, reflecting perfusion to that region, followed by an exponential decrease for the remainder of apnea, as ^13^N_2_ to shunting units is carried away by ongoing blood flow. The magnitude of this decrease is proportional to regional shunt, and robust estimates of regional perfusion and shunt fraction can be derived by applying a mathematical model to the pulmonary kinetics of a ^13^N_2_–saline bolus, measured by PET during apnea (Galletti and Venegas, 2002; O’Neill et al., 2003).

A distillate of the main findings obtained with this technique is:

- Surfactant depletion is accompanied by alveolar derecruitment in dependent regions without major perfusion redistribution toward non-dependent regions. As a result, substantial shunt develops in dependent lung regions in the supine position (de Prost et al., 2011);
- Interventions aimed at recruiting alveoli with increased airway pressure, such as recruitment maneuvers and PEEP, exert two competing effects on the determinants of oxygenation: on one hand they promote re-aeration of derecruited airspaces, thus favoring an improvement of oxygenation, on the other they divert blood flow toward regions that remain derecruited, thus favoring an increase of shunted perfusion and a worsening of oxygenation. The net result of these two effects determines whether oxygenation improves or worsens, and the change in oxygenation can be precisely predicted based on the effect of these interventions on regional shunted blood flow (Musch et al., 2004, 2008);
- Prone positioning restores gas exchange to the dorsal lung in a model of surfactant depletion while only minimally increasing shunted perfusion to the ventral, dependent lung, with consequent dramatic improvement in blood gases (Richter et al., 2005).

Shunt is a functional consequence of edema. Using intravascular tracers that freely diffuse across the endothelium into edematous regions, it is possible to quantify the regional distribution of pulmonary perfusion and extravascular extracellular lung water, i.e., edema fluid. One such technique is based on PET imaging of the pulmonary kinetics of ^15^O-water (H_2_^15^O) to measure regional perfusion and lung water, coupled with PET blood volume scans after inhalation of ^11^C- or ^15^O-carbon monoxide to subtract pulmonary blood volume from regional water (Mintun et al., 1986). Extravascular lung water can then be obtained by subtracting intravascular water from regional water. This technique thus allows determination of regional pulmonary blood flow and extravascular lung water (i.e., edema). Main findings obtained with this technique and other PET techniques that measure regional blood flow are:

Perfusion redistributes away from dependent edematous lung regions in oleic acid-induced lung injury (Gust et al., 1998), to a much greater extent than observed in the lung lavage model (Musch et al., 2004, 2008; de Prost et al., 2011). This redistribution, which acts to preserve arterial oxygenation, is impaired by intravenous endotoxin, thus worsening oxygenation. Because endotoxin blunts hypoxic pulmonary vasoconstriction (Brimioulle et al., 2002; Easley et al., 2009; Fernandez-Bustamante et al., 2009), this observation implies that vascular smooth muscle contraction is responsible for at least part of the observed perfusion redistribution in this model;

Studies in patients with ARDS using the H_2_^15^O technique have also revealed lack of substantive perfusion redistribution away from edematous regions (Schuster et al., 2002), suggesting that hypoxic pulmonary vasoconstriction is, at least to some extent, impaired, similarly to the experimental endotoxin studies (Gust et al., 1998);

Redistribution of perfusion away from injured regions, similar to the oleic acid model, was instead demonstrated after unilateral endobronchial instillation of hydrochloric acid, a model for gastric aspiration, using PET of ^68^Ga labeled microspheres to measure perfusion in rats (Richter et al., 2015).

Edema in ARDS is a consequence of increased pulmonary vascular permeability. This pathophysiological abnormality can be leveraged with PET by imaging the regional distribution of the pulmonary transcapillary escape rate of a radiolabeled protein, such as ^68^Ga-transferrin or ^11^C-methylalbumin, between the intravascular and extravascular space (Schuster et al., 1998). The most consequential insight into ARDS provided by this technique was a dissociation between the topographical distribution of pulmonary vascular permeability increase and that of the resultant edema (Sandiford et al., 1995). Whereas permeability was similarly increased in ventral and dorsal lung regions, extravascular lung density was significantly higher in dorsal, dependent regions, than in ventral, nondependent ones. This finding suggests that even when the inflammatory process involves the entire lung parenchyma, the resultant edema is heterogeneously distributed and tends to concentrate in dependent regions, most likely as a consequence of the effect of gravity.

In addition to PET, a technique that has recently emerged as a potentially useful clinical tool to image the mismatch between pulmonary gas and blood volume distribution is contrast-enhanced dual energy CT. Two CT scans with different radiation energy are simultaneously acquired and then processed to reconstruct the spatial distribution of pulmonary blood volume, a surrogate for pulmonary perfusion (Fuld et al., 2013). Using this technique, Ball et al. (2021) showed that patients with ARDS due to SARS-CoV-2 requiring invasive mechanical ventilation showed both a ventro-dorsal and cranio-caudal gradient in blood volume opposite to that in gas volume, with the result that regions with low gas-to-blood content (i.e., mismatch) increased from ventral to dorsal and from cranial to caudal. Interestingly, however, there were also areas of nonaerated lung that did not appear to contain blood, especially in dependent regions, consistent with pulmonary vascular thrombosis in opacities due to SARS-CoV-2 ARDS.

## Leveraging Anaerobic Metabolism As an Imaging Biomarker For Neutrophilic Inflammation

**Figure 2:**
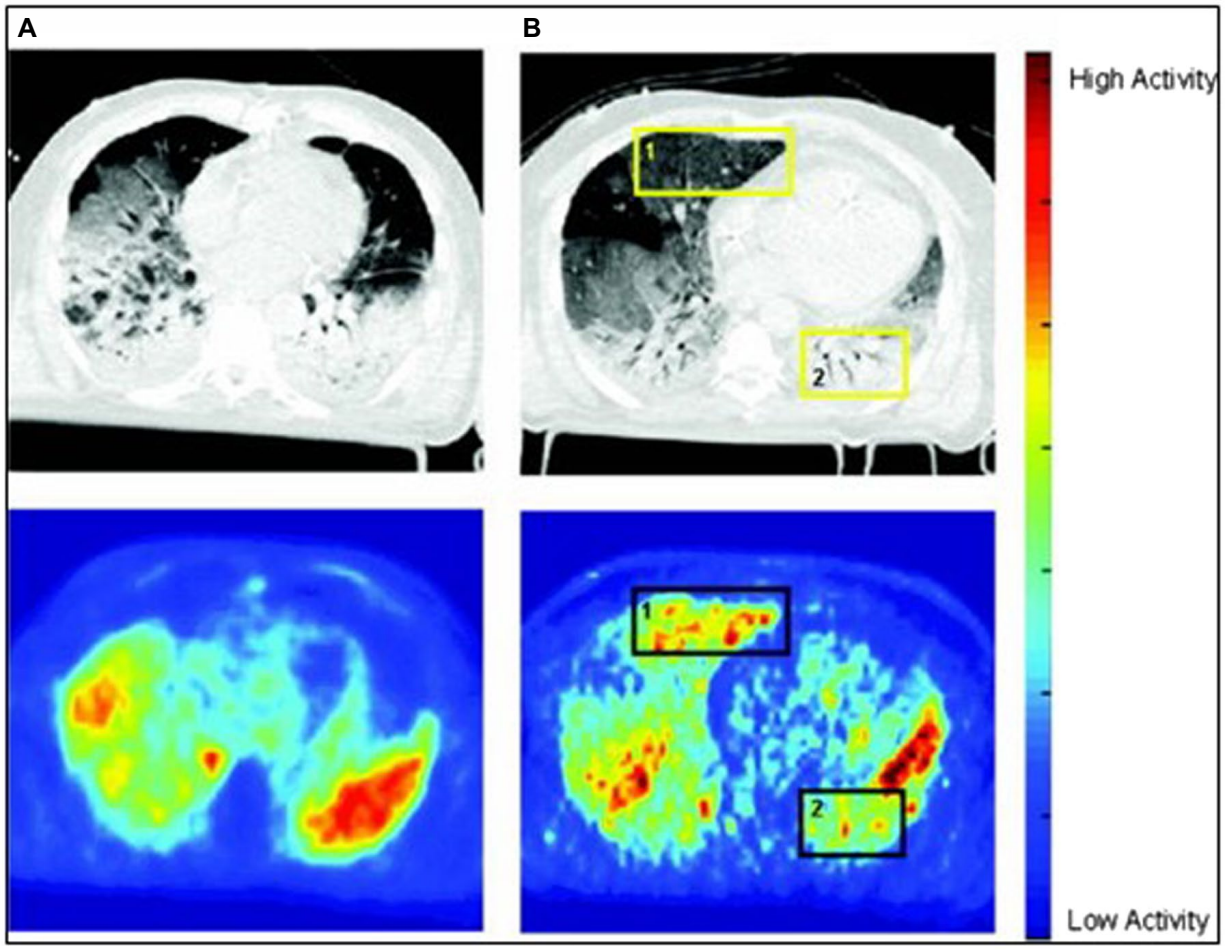
Computed tomography (CT) scans (upper row) and positron emission tomography (PET) scans of [^18^F]FDG uptake (bottom row) in two patients with acute respiratory distress syndrome (ARDS). In patient **A**, [^18^F]FDG uptake is highest in regions of increased lung density on CT, suggesting that these opacities are inflamed and possibly responsible for ARDS. In contrast, in patient **B**, there is a dissociation between [^18^F]FDG signal and density, with increased [^18^F]FDG uptake in nondependent normally aerated regions of both lungs. A hypothesis is that, in this case, the [^18^F]FDG signal mainly reflects ventilator-induced lung injury (VILI), as these are the regions exposed to mechanical ventilation (Reproduced from Bellani et al., 2009).

Acute respiratory distress syndrome and VILI are characterized by a neutrophilic inflammatory response. Neutrophils have very few mitochondria and hence rely primarily on glycolysis for their energy needs. A corollary of this phenotypic trait is that, because glycolysis has low energy yield, activated neutrophils increase their glucose consumption and lactate production disproportionately to other cell types to satisfy their increased energy requirement. This trait can be leveraged by two different metabolic imaging modalities: PET of 2-[^18^F]fluoro-2-deoxy-D-glucose ([^18^F]FDG) and MRI of hyperpolarized [1-^13^C]pyruvate. In the former modality, the positron emitting glucose analog [^18^F]FDG is taken up by cells through facilitative glucose transporters and phosphorylated by hexokinase to [^18^F]FDG-6-phosphate, which cannot proceed further along the glycolytic pathway and thus accumulates in proportion to cellular metabolic rate. Substantive experimental and clinical evidence has established PET measurement of [^18^F]FDG uptake as a tool to noninvasively assess the activation of inflammatory cells in the noncancerous lung (Jones et al., 1994; Chen and Schuster, 2004). In particular, the increase in [^18^F]FDG uptake during VILI was shown to be largely attributable to neutrophils (Musch et al., 2007) and, to a lesser extent, other cell populations such as macrophages and type 2 epithelial cells (Saha et al., 2013). The main insights into the pathophysiology of acute and ventilator-induced lung injury afforded by PET of [^18^F]FDG are:

- Inflammatory cell metabolic activation is an early event in the pathogenesis of lung injury, as increased [^18^F]FDG uptake precedes impairment of pulmonary gas exchange in an acute smoke inhalation model of lung injury (Musch et al., 2014) and development of lung densities in an endotoxemia model with superimposed mechanical ventilation (Wellman et al., 2016);
- The topographical distribution of [^18^F]FDG uptake reflects, at least in part, that of mechanical lung strain. Regions of lung that are most exposed to the biophysical determinants of VILI also show increased [^18^F]FDG uptake (Wellman et al., 2014; Motta-Ribeiro et al., 2018; Retamal et al., 2018). This topographical heterogeneity of [^18^F]FDG uptake is enhanced by the infusion of low-dose endotoxin concomitantly with mechanical ventilation (Costa et al., 2010), a model for clinical sepsis, and reduced by protective ventilation with high PEEP and low tidal volume (de Prost et al., 2013);
- Importantly, regions of lung that present increased [^18^F]FDG uptake on PET also reveal gene expression patterns indicative of activation of specific inflammatory pathways, adhesion molecules, and epithelial and endothelial stretch markers (Wellman et al., 2016; Motta-Ribeiro et al., 2018). In addition, parameters derived from [^18^F]FDG kinetic modeling correlate with specific aspects of the inflammatory response, such as neutrophil infiltration and cytokine expression (de Prost et al., 2014);
- Whereas there is consistent evidence that volutrauma leads to increased pulmonary [^18^F]FDG uptake (Musch et al., 2007; Güldner et al., 2016), results have been conflicting on the role of so called “atelectrauma.” Studies in both animal models (Güldner et al., 2016) and patients (Bellani et al., 2011) have not been able to conclusively demonstrate increased [^18^F]FDG uptake in regions of cyclical tidal recruitment-derecruitment, which are the expected origin of atelectrauma;
- In patients with ARDS, the distribution of lung opacities on CT does not necessarily overlap with that of [^18^F]FDG uptake measured by PET (Bellani et al., 2009; Cressoni et al., 2016). In some patients, the highest [^18^F]FDG uptake occurs in areas that appear consolidated on CT, suggesting that the primary inflammatory process responsible for ARDS is also responsible for the increased PET signal. In other patients, instead, the PET signal is higher in regions with normal aeration. The increased signal in these aerated and ventilated regions could reflect the iatrogenic injury from mechanical ventilation (Figure 2).

Some of the above findings were recently corroborated by metabolic imaging of hyperpolarized [1-^13^C]pyruvate in a hydrochloric acid instillation model of ARDS with superimposed VILI. This MRI method showed increased lactate production, consistent with neutrophilic inflammation, when acid instillation was followed by ventilation with no PEEP. The increased lactate localized predominantly to the dependent dorsal lung in this model, together with increased proton signal intensity consistent with the development of increased lung density from exudate, consolidation, or atelectasis. Protective ventilation with PEEP prevented the increase in lactate signal after acid instillation (Pourfathi et al., 2018).

Main advantages and limitations of the pulmonary imaging modalities presented in this review are summarized in Table 1.

**Table 1:** Main advantages and limitations of lung imaging modalities.

	Advantages	Limitations
EIT	Bedside availability Easy implementation	Poor spatial resolution Limited lung volume sampled
CT	High spatial resolution Speed of acquisition (can image the entire lung volume) Gated acquisition possible	Radiation exposure Limited ability to image biologic processes
PET	Image biologic processes Tracer kinetic modeling Gated acquisition possible	Radiation exposure Lower spatial resolution Longer acquisition times
MR	High spatial resolution Radiation free	Requires hyperpolarized gases to image ventilation Limited ability to image biologic processes

## Conclusion

Over the past 4 decades, substantive evolution in image technology and processing has enabled application of an array of imaging methods to study the acutely injured lung. These methods have yielded fundamental insights into the pathophysiology of VILI and of the gas exchange impairment and inflammatory response of ARDS.

## Author Contributions

The author confirms being the sole contributor of this work and has approved it for publication.

## Funding

Funding for this article was provided by the Department of Anesthesiology and Perioperative Medicine and University of Massachusetts Medical School.

## Conflict of Interest

The author declares that the research was conducted in the absence of any commercial or financial relationships that could be construed as a potential conflict of interest.

## Publisher’s Note

All claims expressed in this article are solely those of the authors and do not necessarily represent those of their affiliated organizations, or those of the publisher, the editors and the reviewers. Any product that may be evaluated in this article, or claim that may be made by its manufacturer, is not guaranteed or endorsed by the publisher.
